# Antifungal alkaloids from *Mahonia* *fortunei* against pathogens of postharvest fruit

**DOI:** 10.1007/s13659-023-00374-3

**Published:** 2023-04-04

**Authors:** Xiao-Na Wang, Zhao-Jie Wang, Yun Zhao, Huan Wang, Mei-Ling Xiang, Yang-Yang Liu, Li-Xing Zhao, Xiao-Dong Luo

**Affiliations:** 1 Key Laboratory of Medicinal Chemistry for Natural Resource, Ministry of Education and Yunnan Province, Yunnan Characteristic Plant Extraction Laboratory, School of Chemical Science and Technology, Yunnan University, Kunming, 650500 People’s Republic of China; 2 State Key Laboratory of Phytochemistry and Plant Resources in West China, Kunming Institute of Botany, Chinese Academy of Sciences Kunming, Kunming, 650201 People’s Republic of China

**Keywords:** *Mahoniafortunei*, Chemical constituents, *Botrytiscinerea*, *Penicilliumitalicum*, Anti-postharvest pathogens

## Abstract

Postharvest pathogens can affect a wide range of fresh fruit and vegetables, including grapes, resulting in significant profit loss. Isoquinoline alkaloids of *Mahonia* *fortunei,* a Chinese herbal medicine, have been used to treat infectious microbes, which might be effective against postharvest pathogens. The phytochemical and bioactive investigation of this plant led to the isolation of 18 alkaloids, of which 9 compounds inhibited the growth of *Botrytis* *cinerea* and 4 compounds against *Penicillium* *italicum*. The antifungal alkaloids could change the mycelium morphology, the total lipid content, and leak the cell contents of *B.* *cinerea*. Furthermore, the two most potent antifungal alkaloids, berberine (**13**) completely inhibited effect on gray mold of table grape at 512 mg L^−1^, while jatrorrhizine (**18**) exhibited an inhibition rate > 90% on grape rot at the same concentration, with lower cytotoxicity and residue than chlorothalonil, which suggested that ingredients of *M.* *fortunei* might be a low-toxicity, low-residue, eco-friendly botanical fungicide against postharvest pathogens.

**Graphical Abstract:**

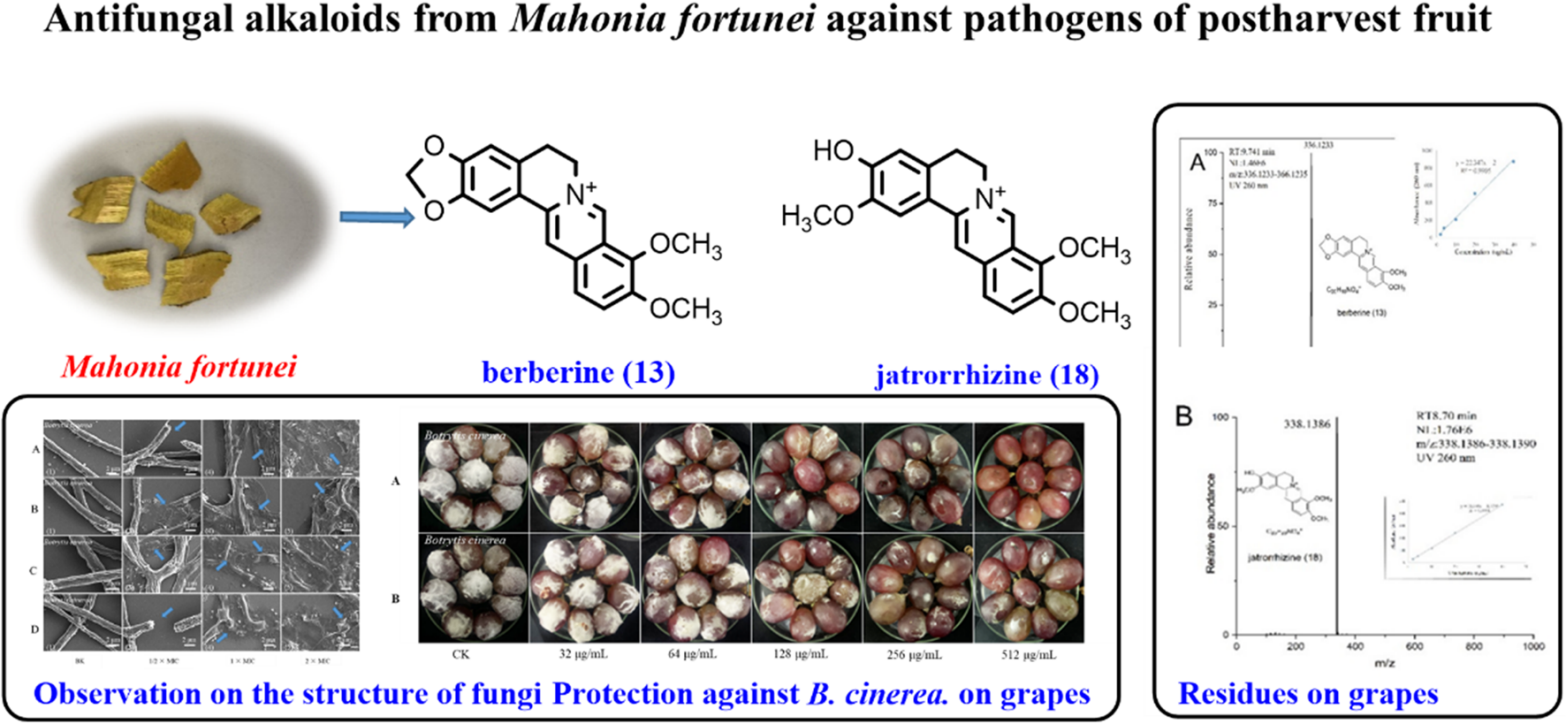

**Supplementary Information:**

The online version contains supplementary material available at 10.1007/s13659-023-00374-3.

## Introduction

The major factor affecting the yield of fresh fruit and vegetables is postharvest fungal disease, such as *Botrytis* *cinerea* and *Penicillium* *italicum* [1], which usually endangers the three critical periods of flowering, maturation and storage of grapes, tomatoes, citrus and other fruit and vegetables. This results in loss of berry shedding and causes rot and loss during storage and transportation [2], accounting for 20–30% of crop losses during postharvest annually and even up to 50% in severity [3]. To date, application of chemical fungicides and low-temperature are widely used in controlling postharvest diseases. However, the characteristics of toxicity to animals, and the difficulty in degrading chemical fungicides have evoked environmental concerns all over the world, and their residues in fruit and vegetables have led to the ban. Furthermore, resistance to fungicides has been widely developed, although fungicides such as dimethylimide can prevent the damage of *P.* *italicum* and *B.* *cinerea* to vegetables and fruit [4]. A large number of botanical fungicides are generally regarded as green, pollution-free and low-toxic substances, and substitutes of fungicides can be sought from plants to control postharvest diseases of fruit and vegetables [5].

Isoquinoline alkaloids were a group of natural products that were primarily isolated from *Papaveraceae*, *Berberidaceae* and *Ranunculaceae* and were known for their antitumor, antifungal and analgesic activities [6]. Among berberine has antifungal activity against *Colletotrichum* *capsici* [7], while sanguinarine and chelerythrine inhibit *Alternaria* *alternate*, *Curvularia* *lunata* and *Valsa* *mali* [8]. *Mahonia* *fortunei*, widely distributed in America and Asia, is a traditional Chinese medicine with abundant isoquinoline alkaloids [9, 10]. It had pharmacological effects on *Staphylococcus* *aureus,* *Candida* *albicans* [11, 12], and *Xanthomonas* *oryzae* [13]. However, the lack of reports on its extract and compounds in phytopathogens control encouraged us to test antifungal bioactivity of *M.* *fortunei* against *P.* *italicum* and *B.* *cinerea*. As a result, its extracts showed inhibitory effects on two plant pathogens, and then the bio-guided isolation led to the identification of anti-fungal alkaloids from *M.* *fortunei*. Finally, antifungal effects of bioactive isoquinoline were examined in vitro and in vivo and the possible antifungal mechanism was studied.

## Materials and methods

### Experimental

Column chromatography (CC) was performed with silica gel (200–300 mesh), RP-C18 **(**40–63 μM**)** Sephadex LH-20 and Agilent 1260 Infinity II (USA) semipreparative RP-C18. Compounds were identified by ESI–MS spectra (Agilent 1290/6545Q-TOF, USA) and NMR spectra (BRUKER Avance III, 400 MHz, Switzerland). Ethyl acetate, petroleum ether, ethanol, chloroform, dichloromethane, methanol, acetone, Tween 80 and glucose were obtained from Tianjin Chemical Reagents (Tianjin, China). Chlorothalonil and MTT were obtained from Macklin Reagents (Shanghai, China).

### Fungal pathogen and fruit

Pure cultures of *B.* *cinerea* and *P.* *italicum* in this study were from the State Key Laboratory for Conservation and Utilization of Bio-Resources in Kunming, Yunnan Province, P. R. China. The fungal strains were stored on potato dextrose agar (Medium) (PDA) slants and kept out of light at 4 °C. The fungal spore suspension was harvested by submerging the PDA plate with 5 mL of potato dextrose broth (PDB) and filtering, and adjusted to 1 × 10^5^ CFU mL^−1^ by a hemocytometer. Grapes (*Vitis* *vinifera*) with uniform size, maturity and color and no obvious disease were purchased from the fruit supermarket of Yunnan University.

### Plant materials

*M.* *fortunei* was purchased from the Wanyao Store, Kunming, Yunnan Province, China. The plant was identified by Professor Li-Xing Zhao from Yunnan University. The specimen (No. YD-281) was deposited in the Key Laboratory of Medicinal Chemistry for Natural Resource, Ministry of Education and Yunnan Province, School of Chemical Science and Technology, Yunnan University, Kunming, P. R. China.

### Extraction and isolation

The pulverized stems *of* *M.* *fortunei* (10 kg) were extracted by refluxing with 75% ethanol solution (2 h × 4) at 60 °C. The obtained crude extract was diluted in HCl (pH 2–3). Next, the solution was filtered, the pH of the filtrate was adjusted to 9–10 with ammonia water, and it was continuously distributed with ethyl acetate to obtain non-alkaloid, small polar alkaloids and large polar alkaloids moieties [14]. The small polar alkaloids moiety (84 g, MIC _*B.cinerea*_ = 200 mg/L, MIC _*P.italicum*_ = 200 mg/L) was chromatographed for analysis using a silica gel column and CHCl_3_-MeOH gradient (45:1, 30:1, 15:1, 5:1, 0:1 v/v) to obtain seven fractions (Frs. I–VII), the detailed method was presented in the Additional file 1.

### Antifungal activity test in vitro

#### Inhibition of spore germination

The minimum inhibitory concentrations (MIC) and minimum fungicidal concentrations (MFC) of the compounds were determined by the double dilution method in 96-well plates [15]. Briefly, the initial series concentrations of the components and compounds were added separately, and the volumes were 50 μL. Afterwards, 50 μL of fungal spore suspension (1.0 × 10^5^ CFU mL^−1^) was added to obtain five concentration gradients of compounds (128, 64, 32, 16, 8, 4, 2 mg L^−1^). Finally, 96-well plates were cultured in the darkness at 24 °C for 48 h, and the spore germination was measured under a microscope. Those without any germination were known as the MIC [16]. An aliquot (10 μL) of each well at concentrations above the MIC was inoculated on PDA and cultured at 24 ℃ for 48 h. The wells without any germination were known as the MFC. A positive control (chlorothalonil), blank control were incubated in PDB containing 1% DMSO [17]. The experiment was repeated 3 times.

#### Inhibition on the mycelial growth

Two major bioactive compounds berberine (**13**) and jatrorrhizine (**18**) were mixed into the PDA culture medium to obtain concentration ranges (512, 256, 128, 64 and 32 mg L^−1^), and then 5 mL of PDA was evenly transferred into sterilized petri dishes (inner diameter 60 mm) [18]. A mycelium plug (5 mm in diameter) was inoculated on the PDA culture dish in the center of the dish and each group was repeated 3 times. The culture dishes were sealed with paraffin film and cultured in the darkness at 24 ℃ for 48 h. The radial growth of hyphae was measured and the inhibitory effect was expressed as EC_50_ (50% concentration of the maximum effect). The EC_50_ value was calculated based on the relationship between the concentrations of the compounds and mycelial radial growth. First and foremost, the following formula was used to determine the relative inhibition rate (%) of mycelium growth [19]:

$$\mathrm{Mycelial \, growth \, inhibition \, }(\mathrm{\%})=(\mathrm{C}-\mathrm{T})/\mathrm{C}\times 100$$

Here C and T were the mean diameter (mm) of mycelia of the blank control and treatment groups, respectively. Next, the inhibition rate was converted into a probability value (Y) and the compound concentrations (X) were log-transformed. Then, linear regression (Y = a + bx) was performed to estimate the coefficient (R^2^) from the computer-generated compound concentration and relative inhibition plots.

#### Scanning electron microscopy (SEM)

The spore suspensions of *B.* *cinerea* and *P.* *italicum* were placed in PDB medium, and cultured for 2 d on a shaker at 24 ℃ and 150 g [20]. The mycelium was collected and washed three times with phosphate buffered solution (PBS, pH = 6.8). Subsequently, compounds were added to treat *B.* *cinerea* at concentrations of 0, 1/2 × MIC, 1 × MIC and 2 × MIC, with three replicate wells set. After 120 min of treatment, the mycelium were fixed with 4% paraformaldehyde for 12 h at 4 ℃. Next, the samples were shifted to a string of ethanol solutions (30%, 50%, 70% and 90%) for 15 min every time, and then treated with 100% ethanol for 20 min. Thereafter, the treated mycelium was subjected to vacuum freeze-drying and SEM observation.

#### Determination of release of cell constituents

The release of cellular components was determined by gauging the absorbance (260 nm) of a *B.* *cinerea* suspension [21]. In brief, the mycelium of *B.* *cinerea* from 500 μL PDB of three-day-old was collected by centrifugation at 2950 g for 20 min, washed with PBS 3 times, and resuspended in 2 mL PBS. In the presence of 1 × MIC and 2 × MIC compounds, respectively, they were incubated on a shaking table (150 g) at 24 ℃ for 0, 60, 180 and 240 min. Subsequently, 2 mL samples were collected, centrifuged at 12,000 g for 2 min, and the absorbance at 260 nm was measured.

#### Determination of total lipid content

The total lipid content of cells was established by the vanillin phosphate method [22]. The mycelium of three-day-old *B.* *cinerea* from 25 mL of PDB was collected and incubated with different concentrations (0, 1 × MIC and 2 × MIC) of the compounds at 24 ℃ for 4 h. The mycelium was dried with a vacuum freeze dryer for 8 h, and 0.01 g of the dried mycelium was shaken vigorously in 4.0 mL of methanol-chloroform-water mixed solvent (2:1:0.8, v/v) for 30 min. The lipid-containing lower phase was mixed well with 0.2 mL of the salt solution and centrifuged at 4000 g. Aliquots of 0.1 mL of the lipid mixture were then moved to new tubes, and 0.2 mL of sulfuric acid was added, and heated in a boiling water bath for 10 min. Thereafter, 3 mL of vanillin phosphate was added and reacted for 10 min. The absorbance was measured at 520 nm and the total lipid content was calculated based on the standard curve for cholesterol.

### Protection against *B. cinere*a on grapes

First, the grapes were soaked in 75% ethanol for 3 min and then washed with distilled water to remove the ethanol residue [23]. Second, the fruit were soaked in compounds including 1% Tween 80 at concentrations of 512, 256, 128, 64 and 32 mg L^−1^ for 3 min. Finally, a sterile needle was used to puncture three points at the equator of each fruit, and afterwards, 10 μL of the fungal spore suspension diluted to 1.0 × 10^6^ CFU mL^−1^ was applied to the wound. The treated fruit were stored in a humidified 1 L airtight container at 24 °C, with 10 fruit per treatment. When the pathogen absolutely overspread the surface of the blank control fruit, the damaged area of the treated fruit were measured with Vernier caliper. The process was performed 3 times. The decay rate was calculated based on the following formula:

$$\mathrm{Ratio \, of \, decay \,}(\mathrm{mm}^{2})=\uppi \times \mathrm{a}\times \mathrm{b}$$

$$\mathrm{Inhibition \, rate }\left(\mathrm{\%}\right)=\left(\mathrm{C}-\mathrm{T}\right)/\mathrm{C}\times 100$$

Here, a and b represent act the transverse and radial damage radii (mm) of the inoculum, respectively, and C and T represent the average proportion of rotting lesions (mm^2^) in the control group and treatment groups, respectively.

### Cytotoxicity assay

The cytotoxic effects of compounds on HacaT cells from Yunnan University of Chinese Medicine were detected by the MTT method [24]. The cell line was incubated in DMEM with the addition of fetal bovine serum (10%) and penicillin and streptomycin (1%) containing carbon dioxide (5%) at 37 ℃. A total of 5 × 10^3^ cells/well were added to a 96-well plate for culture for 24 h. Then, 16 mg L^−1^, 32 mg L^−1^ compounds and chlorothalonil were added and cultured for 72 h, with three repeat wells. The supernatant was collected by centrifugation at 1500 g for 15 min and incubated with MTT solution (10%) for 3 h. Next, the precipitate was dissolved in DMSO (150 μL) and the absorbance at 595 nm was measured.

### Residue of sample in grapes

Extraction and purification of grape samples was performed by the QuEChERS method [25], with slight modifications. Each grape was treated with 0.2 mL 512 mg L^−1^ berberine, jatrorrhizine and chlorothalonil. Fifteen gram grape samples were placed into polypropylene centrifuge tubes, 15 mL of acetonitrile and 6 g of MgSO_4_ were added, and the samples were centrifuged at 5000 g for 3 min. Next, 2 mL of extract and 3 + 1 (w/w) MgSO_4_-N-propyl ethylenediamine solid phase adsorbent (200 g L^−1^ extract) were mixed and centrifuged. The final extract was analyzed by LCMS.

## Results

### Compounds from the stems of *M. fortunei*

**Fig. 1:**
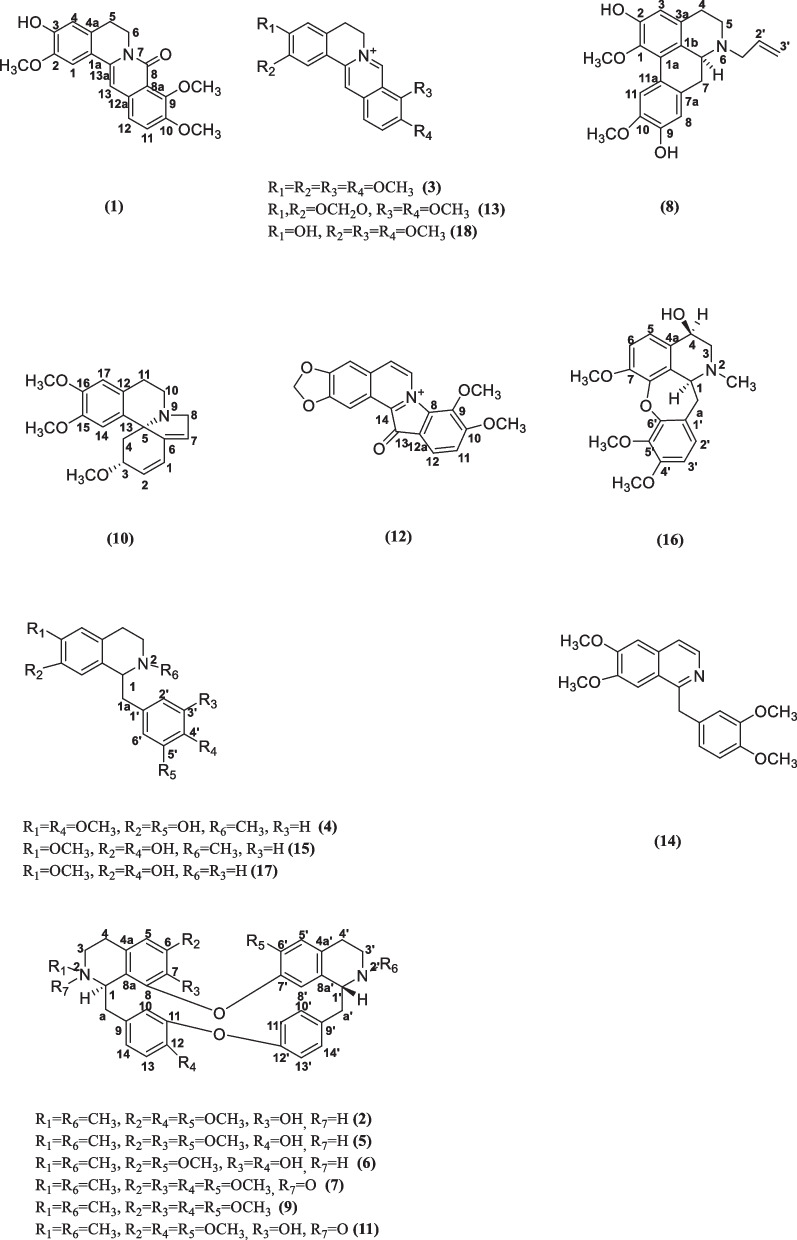
Structure of compounds

Eighteen known alkaloids sinotumine I (**1**) [26], limacine (**2**) [27], palmatine (**3**) [28], reticuline (**4**) [29], berbamine (**5**) [30], obamegine (**6**) [30], fenfangjine A (**7**) [31], N-allyllaurolitsine (**8**) [32], isotetrandrine (**9**) [33], erysotrine (**10**) [34], fenfangjine P (**11**) [35], coptisonine (**12**) [36], berberine (**13**) [28], papaverine (**14**) [37], N-methycoclaurine (**15**) [38], ( +)-4-Hydroxysarcocapnine (**16**) [39], coclaurine (**17**) [40], and jatrorrhizine (**18**) [28] were isolated from *M.* *fortunei*, and their structures were identified by comparison with the literatures (Fig. 1).

### Antifungal assays in vitro

#### Inhibition of spore germination

The MICs and MFCs were carried out on the anti-*B.* *cinerea* and *P.* *italicum* bioactivities of all compounds. The results showed that palmatine (**3**), berbamine (**5**), berberine (**13**) and jatrorrhizine (**18**) inhibited the spore germination of *P.* *italicum*. The spore germination of *B.* *cinerea* could be inhibited by sinotumine I (**1**), limacine (**2**), palmatine (**3**), reticuline (**4**), berbamine (**5**), fenfangjine A (**7**), erysotrine (**10**), berberine (**13**) and jatrorrhizine (**18**). Compounds **1** and **13** had the same MIC and MFC values, which were 16 mg L^−1^ and 128 mg L^−1^, respectively. Among them, the anti-*B.* *cinerea* bioactivities of compounds **1**, **2**, **3**, **4**, **5**, **7** and **10** were reported for the first time (Table 1).

**Table 1:** The MICs and MFCs of the individual compounds of *M.* *fortunei* against spore germination of *B.* *cinerea* and *P.* *italicum*

Compounds	*Botrytis* *cinerea*		*Penicillium* *italicum*	
	MIC mg L^−1^	MFC mg L^−1^	MIC mg L^−1^	MFC mg L^−1^
1	16	128	–	–
2	32	128	–	–
3	32	128	64	> 128
4	32	128	–	–
5	32	128	32	> 128
7	32	128	–	–
10	32	> 128	–	–
13	16	128	32	> 128
18	32	128	64	> 128
Chlorothalonil	8	32	4	16

#### Inhibition on the mycelial growth

**Fig. 2:**
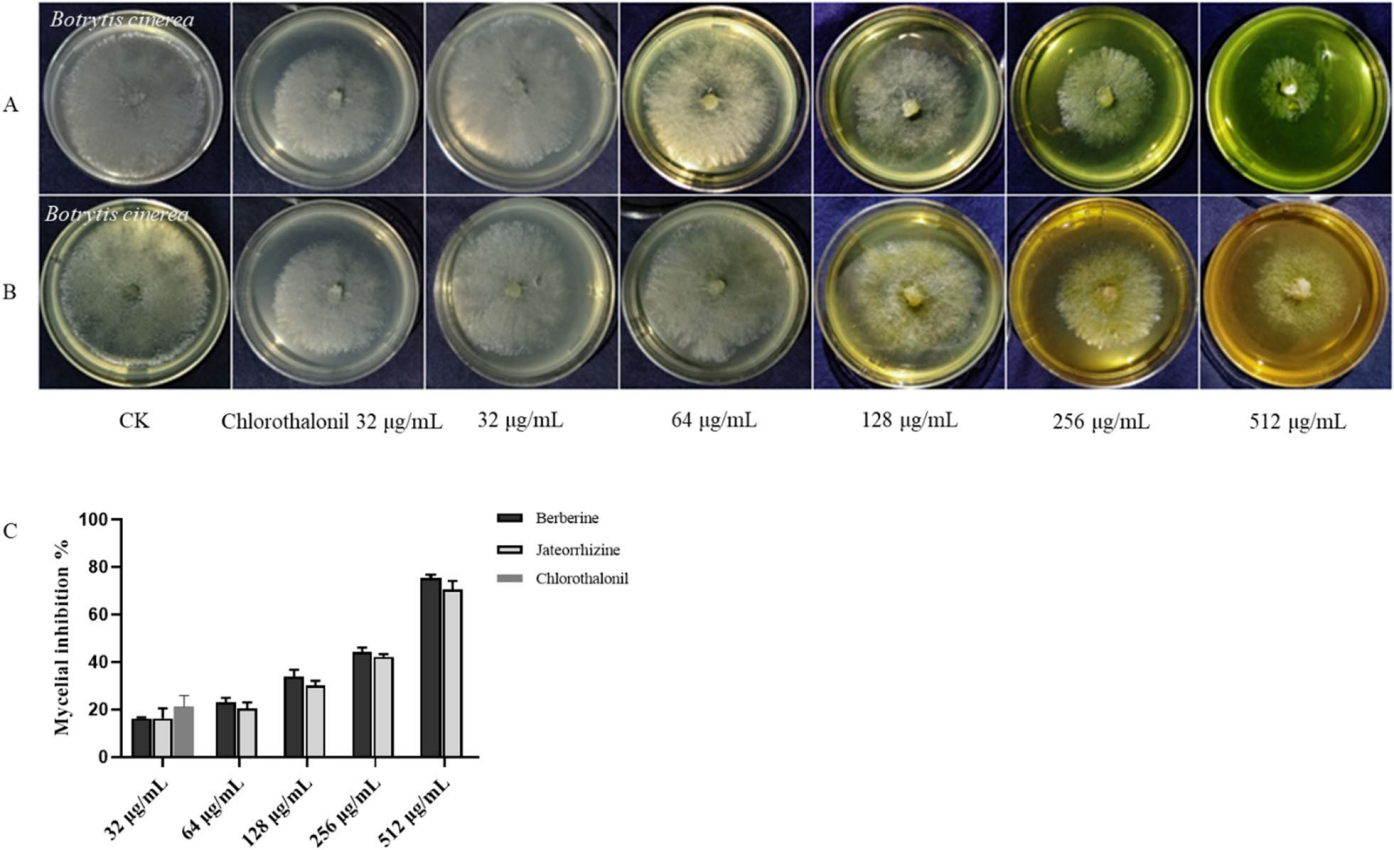
The compounds inhibited mycelial growth of *B.* *cinerea*. Treatment with (**A**) berberine; (**B**) jateorrhizine; (**C**) inhibition rate of mycelia growth

The antifungal activities and EC_50_ values of the compounds against *B.* *cinerea* in vitro were shown in Fig. 2 and Table 2. The compounds against hyphal growth of *B.* *cinerea* presented a quantity-effect relationship. At a concentration of 32 mg L^−1^, berberine (**13**) and jatrorrhizine (**18**) had similar inhibitory effects on mycelial growth, and the inhibition rate was only approximately 15%. However, at 512 mg L^−1^, the inhibition rate of berberine was 75%.

**Table 2:** EC_50_ values of the compounds inhibited mycelial growth of *B.* *cinerea*

Compound	Regression equation	R^2^	EC_50_ (mg L^−1^)	95% Confidence intervals (mg L^−1^)
Berberine	Y = 1.301*X + 1.962	0.9399	216	145.60–322.40
Jateorrhizine	Y = 1.226*X + 2.019	0.9245	270	154.91–472.03

#### Scanning electron microscopy (SEM)

**Fig. 3:**
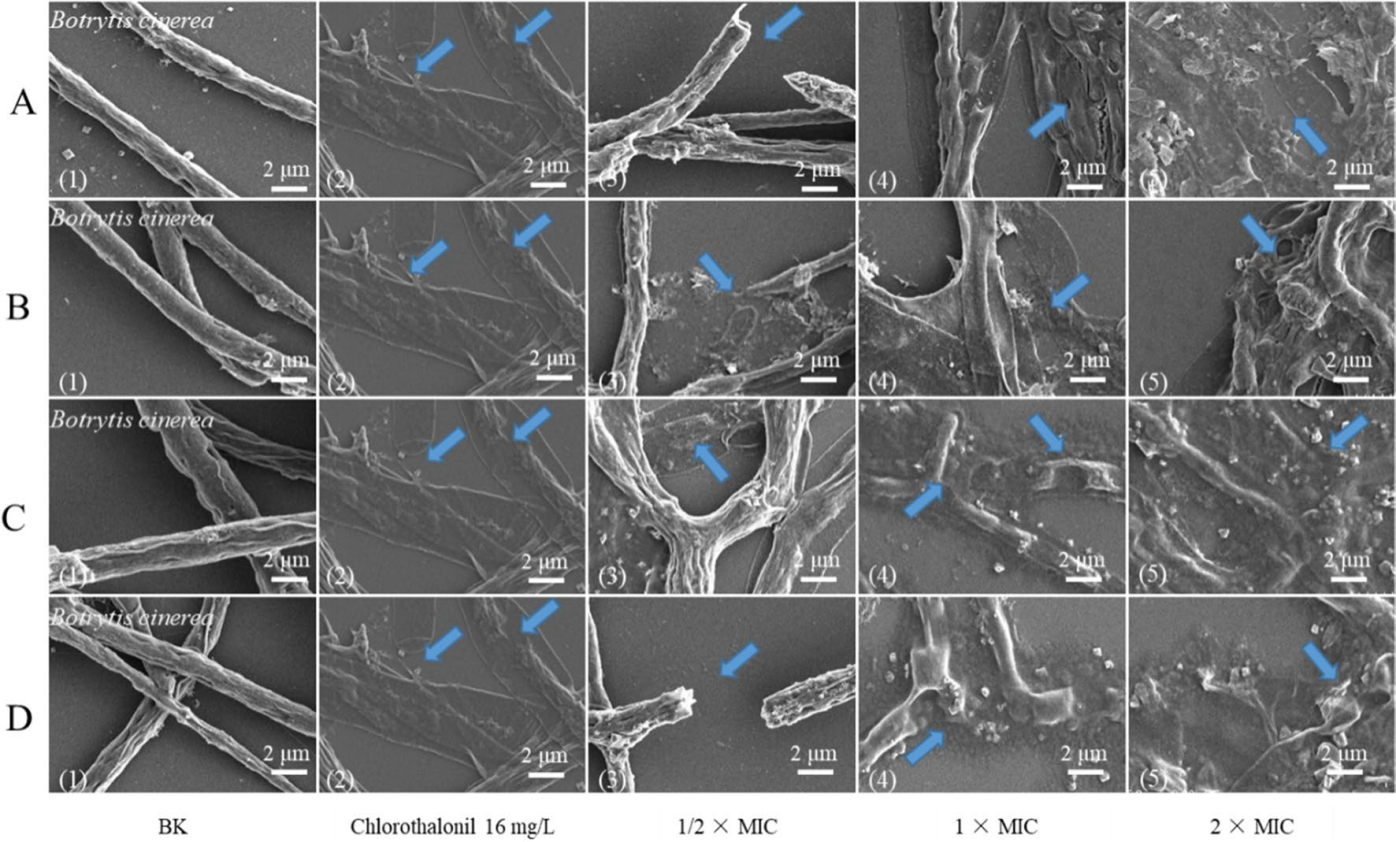
SEM photos of *B.* *cinerea* untreated mycelium (**A1**–**D1**); mycelia treated with sinotumine I (**A**), reticuline (**B**), berberine (**C**) and jatrorrhizine (**D**) at 1/2 × MIC, 1 × MIC, 2 × MIC (**A3**–**D5**); mycelium treated with chlorothalonil at 16 mg L^−1^ (**A2**–**D2**). The blue arrow represents the observed morphological change

The mycelia of *B.* *cinerea* and *P.* *italicum* were treated with the active compounds at concentrations of 1/2 × MIC, 1 × MIC and 2 × MIC for 3 h, respectively (Fig. 3). The morphological changes of mycelium of *P.* *italicum* were shown in the Additional file 1. The untreated mycelium surface of *B.* *cinerea* (Fig. 3A1, D1) presents a typical linear uniform, complete, smooth and regular surface. After contact with 1/2 × MIC jateorrhizine (16 mg L^−1^) and berberine (8 mg L^−1^) (Fig. 3B3, C3), most hyphae became irregular and shriveled. Similarly, after treatment with the concentration of 2 × MIC sinotumine I (32 mg L^−1^) (Fig. 3A5), the morphology of fungal hyphae changed and deformed obviously, even leading to leakage of contents. After exposure to the positive control drug chlorothalonil at 16 mg L^−1^ (Fig. 3A2), the mycelium morphology of fungi became concave and irregular and contracted. This result indicated that the fungal toxicity of these compounds may be achieved by destroying cell membranes and further blocking cell growth.

#### Determination of release of cell constituents and total lipid content

**Fig. 4:**
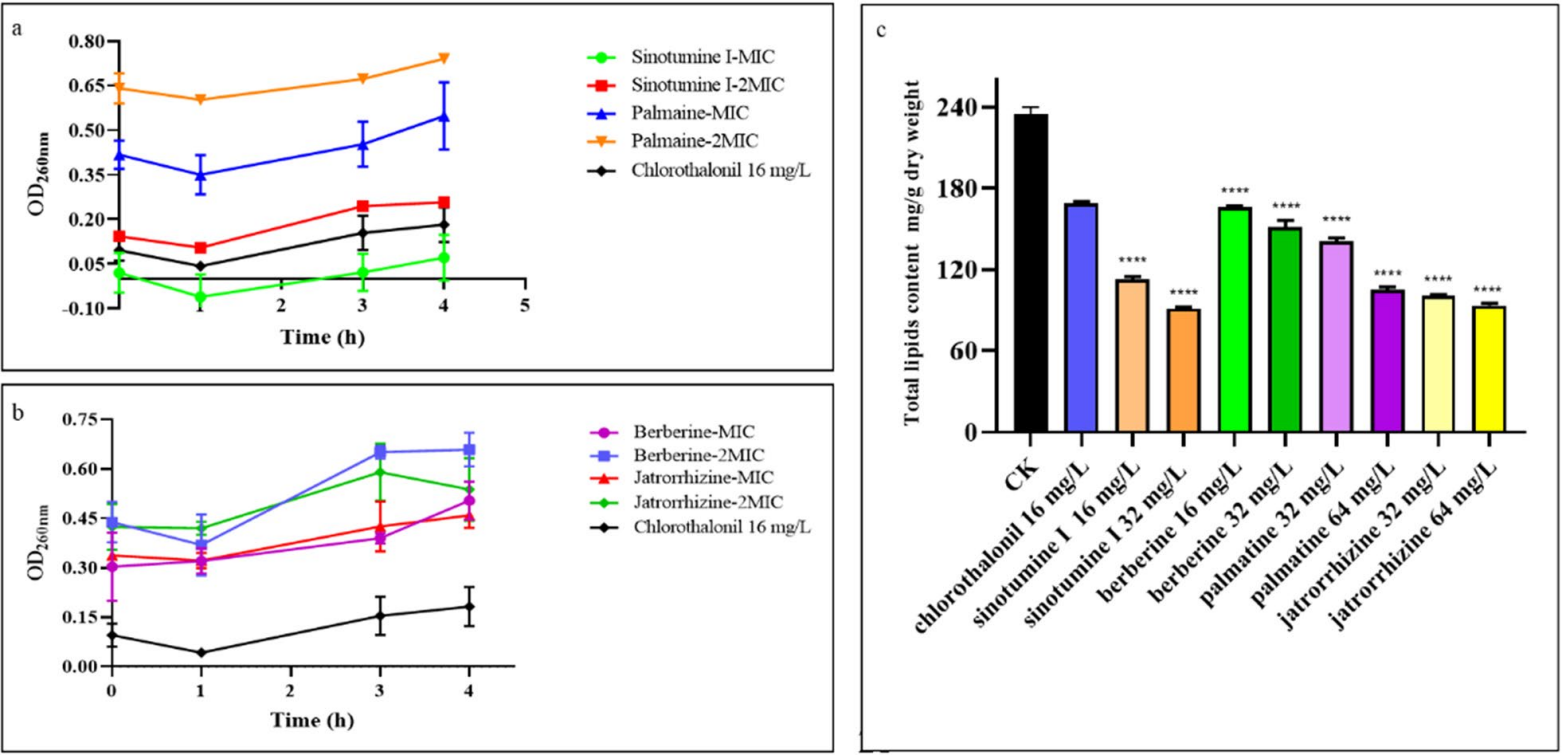
Effects of compounds at MIC and 2 MIC on the release of 260 nm absorption substances of *B.* *cinerea* treated with sinotumine I and palmatine (**a**); berberine and jatrorrhizine (**b**); total lipid content of *B.* *cinerea* cells in the presence of different concentrations of the compounds sinotumine I, palmatine, berberine and jatrorrhizine (**c**). Data displayed were the average value of the aggregated data. ****P < 0.0001 versus CK

Although the OD_260_ of the suspension treated with active compounds decreased slightly after 60 min of exposure, the absorbance of the suspension changed significantly in the subsequent cell treatment. The OD_260_ of the suspension treated with jatrorrhizine at 1 × MIC (32 mg L^−1^) for 180 min was 0.43, which was obviously higher than that of the control group (0.34) (Fig. 4b). After 180 min of exposure, the OD_260_ values of sinotumine I at 2 × MIC (32 mg L^−1^) and palmatine at 1 × MIC (32 mg L^−1^) were 0.24 and 0.45, respectively, slightly higher than those of the control group (0.14 and 0.42). After positive chlorothalonil treatment for 180 min, the OD_260_ value was 0.15, slightly higher than that of the control group (0.09).

The effect of the compounds on the total lipid content of *B.* *cinerea* was shown in Fig. 4c After incubation with compound sinotumine I (**1**) at 1 × MIC (16 mg L^−1^) for 4 h, the total lipid content was 112.7 mg g^−1^ dry weight, which was remarkably lower than that of the control group (234.9 mg g^−1^ dry weight) and chlorothalonil treatment group (168.9 mg g^−1^ dry weight). The total lipid content (151.2 mg g^−1^ dry weight) of the cells after *B.* *cinerea* exposure to berberine (**13**) at 2 × MIC (32 mg L^−1^) was also lower than that in the control group. Palmatine (**3**) and jatrorrhizine (**18**) both reduced the cell lipid content after treatment with *B.* *cinerea* cells.

### Protection against *B. cinerea* on grapes

**Fig. 5:**
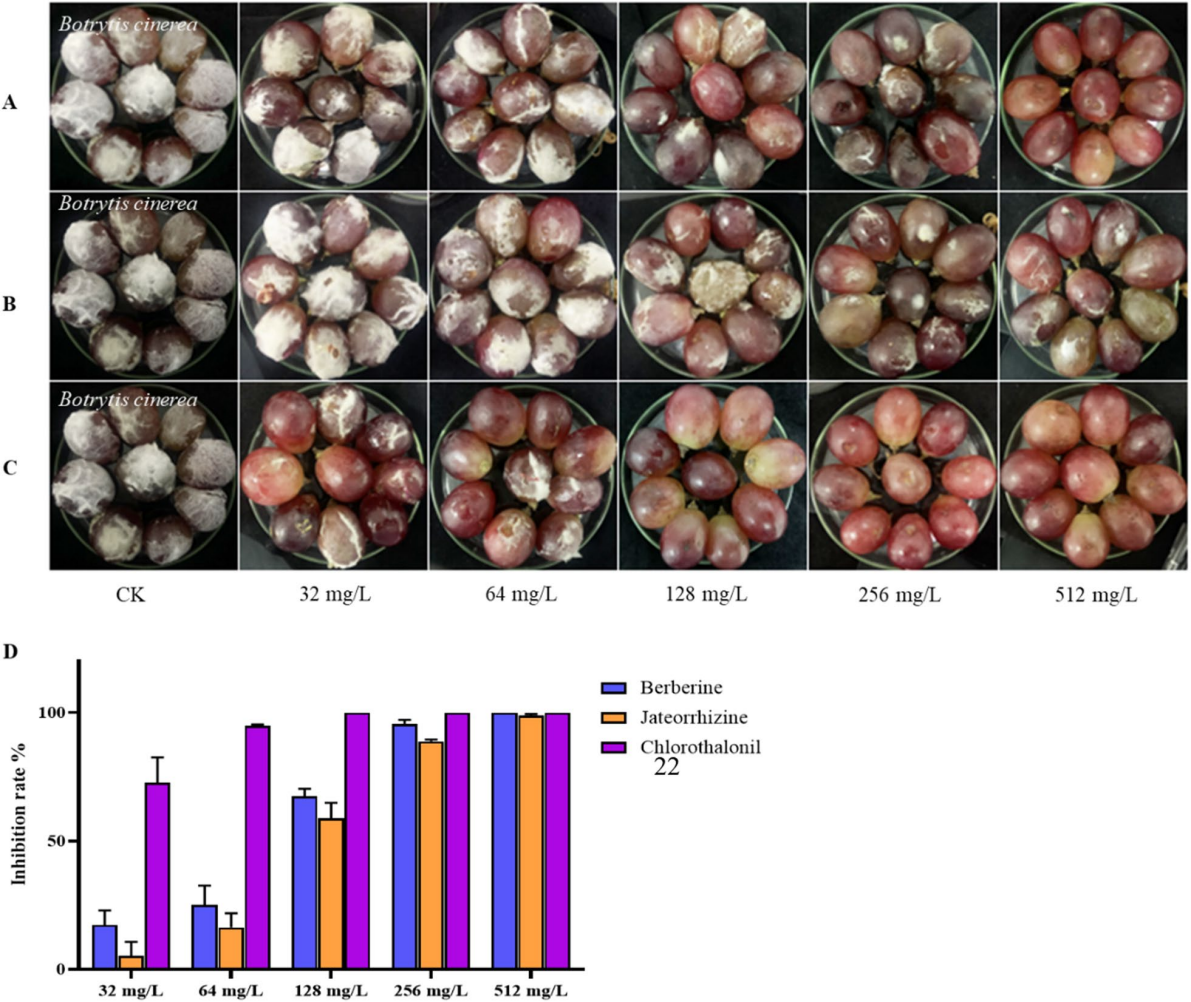
Prevention of against *B.* *cinerea* of grapes with two compounds after 10 d. Treatment with (**A**) berberine; (**B**) jateorrhizine; (**C**) chlorothalonil; (**D**) inhibition rate on grape rot

The antifungal effects of the alkaloid moiety of *M.* *fortunei* on grapes were shown in the Additional file 1. With berberine (**13**) at 128 mg L^−1^, there was a 50% reduction in the decay of grapes compared with untreated samples. At 512 mg L^−1^, berberine completely inhibited grapes rot. Jatrorrhizine (**18**) produced a 99% reduction in the decay of grapes at 512 mg L^−1^ (Fig. 5). This meant that berberine and jatrorrhizine could inhibit gray mold on grapes.

### Residue of sample in grapes

**Fig. 6:**
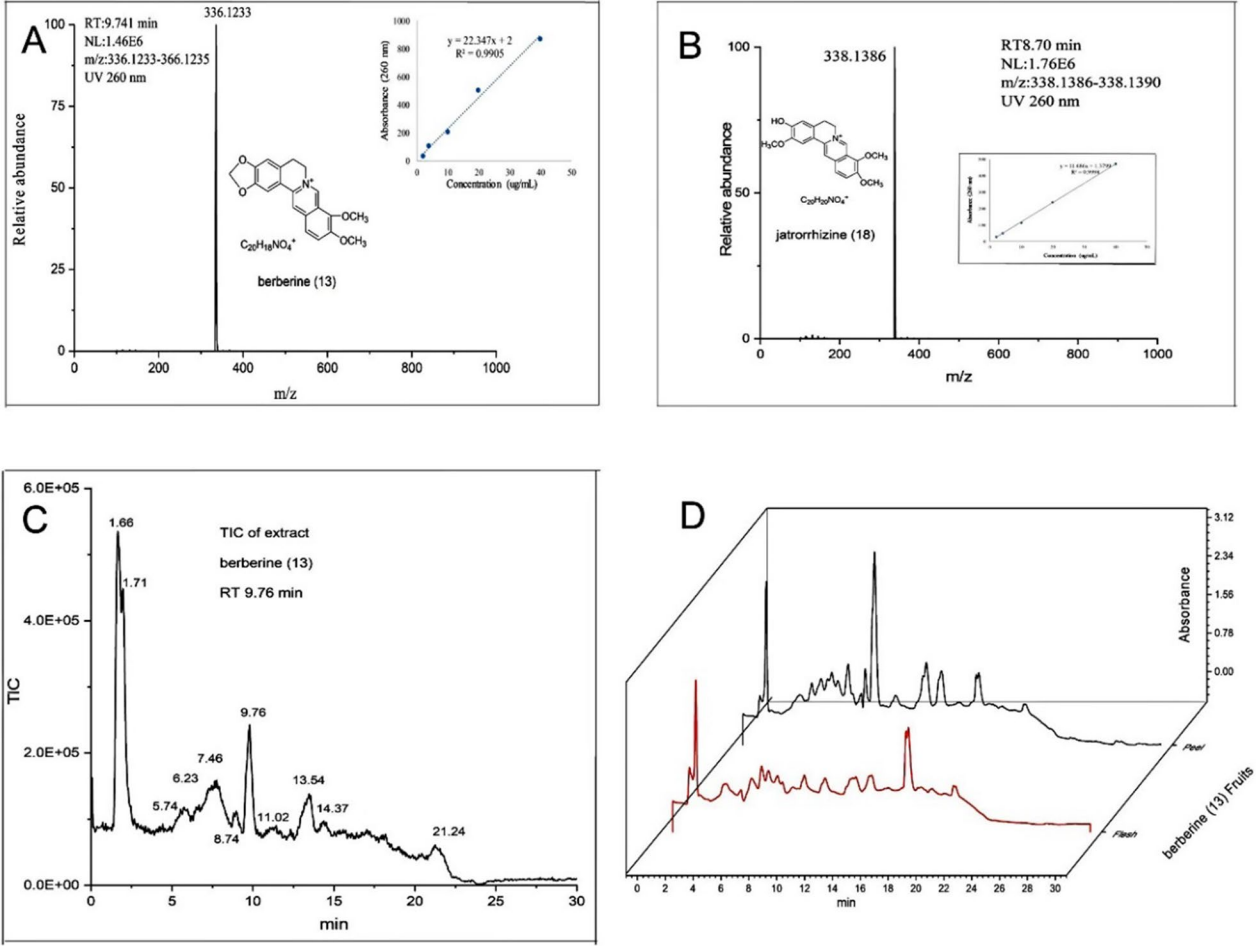
UHPLC/ESI Q-Orbitrap MS chromatograms and spectra: qualitative compounds by LC–MS characteristics berberine (**A**); jatrorrhizine (**B**); total ion chromatogram of crude fruit extracts (**C**); UV (260 nm) absorption curve of crude fruit extracts (**D**)

The residues of grapes treated with berberine and jatrorrhizine and chlorothalonil as positive controls were detected by LC–MS (Fig. 6, Table 3). There was almost no residue in the peel and flesh of grape treated with jatrorrhizine, and the residue was 0.004% (the residue was the total residual as a percentage of the total amount used). Berberine residue was found in both grape peel and grape flesh, but its toxicity was relatively low. The residue of chlorothalonil on grape peel was 0.35%, much higher than the jatrorrhizine residue.

**Table 3:** HPLC/MS detection sample residue experiment

	Berberine (**13**)	Jatrorrhizine (**18**)	Chlorothalonil (B)
Peel (residue)	0.98%	< 0.004%	0.35%
Flesh (residue)	0.06%	< 0.004%	0.004%
	Y = 22.347x + 2	Y = 11.686x + 1.3799	Y = 17.046x-5.6589
R^2^	0.9905	0.9998	0.9923

## Discussion

One of the most potent bioactive compounds berberine, produced on a large scale in industry with antibacterial, anti-cancer [41], anti-inflammatory [42] and obesity prevention bioactivity [43], showed antifungal bioactivity in vitro against *B.* *cinerea* [44]. Now, the protective effect against *B.* *cinerea* on grapes of berberine and jatrorrhizine in vivo further verified their prevention and treatment of postharvest gray mold for the first time. Moreover, one of the main advantages of *M.* *fortunei* is that its residue on the product is very low and has low toxicity, which will facilitate the development of alternatives to synthetic fungicides. And then SEM micrographs indicated that the bioactive alkaloids of *M.* *fortunei* could alter cellular morphology and destroy membrane of *B.* *cinerea*, and the hyphae even collapsed and fractured after incubation with higher concentrations of bioactive alkaloids. In fact, the integrity of the cytoplasmic membrane is the key factor in fungal growth, material exchange and information transmission between cells and the outside world, as well as in maintaining the stability of the intracellular environment [45]. Therefore, damage to the membrane can lead to the leakage of cell contents [46]. Measuring the release of cellular material is an indicator of cell lysis, and significant leakage of cytoplasmic matter often demonstrates severe and irreversible impairment to the cytoplasmic membrane and plasma membrane [47]. The decrease in lipid content also suggests that membrane stability may be reduced [48]. Here, an obvious leakage of the contents of the mycelia was observed after treatment. Further exposure of *B.* *cinerea* to the bioactive alkaloids resulted in a decrease in lipid content, the main component of biofilm. Therefore, we infer that it may cause damage to the cell membrane system and show antifungal activity.

## Supplementary Information

**Additional file1:** Separation process and NMR information of 18 compounds; Antifungal activity of small polar alkaloids from *M.* *fortunei*; Morphological changes of *P.* *italicum* mycelium treated with active compounds; Cytotoxicity assay.

## Data Availability

Relevant data from this study had been submitted to the Science Data Bank. (DOI:10.57760/sciencedb.06816).

## Abbreviations

CC: Column chromatography
MIC: Minimum inhibitory concentration
MFC: Minimum fungicidal concentration
PBS: Phosphate buffered solution
SEM: Scanning electron microscopy
TIC: Total ion chromatograms
PDA: Potato dextrose agar
PDB: Potato dextrose broth
MTT: 3-(4,5-Dimethylthiazole-2-yl)-2,5-diphenyltetrazolium-bromide
NMR: Nuclear magnetic resonance spectroscopy
MPLC: Medium-pressure liquid chromatography
