# Supplementary material for: Antifungal alkaloids from Mahonia fortunei against pathogens of postharvest fruit
Source: Nat Prod Bioprospect. 2023 Apr 4;13(1):10. doi: 10.1007/s13659-023-00374-3 (PMC10070590; doi:10.1007/s13659-023-00374-3)
Supplement: Supplementary file 1 — Additional file1: Separation process and NMR information of 18 compounds; Antifungal activity of small polar alkaloids from M. fortunei; Morphological changes of P. italicum mycelium treated with active compounds; Cytotoxicity assay. [file 13659_2023_374_MOESM1_ESM.docx]

**Supporting Information**

**Antifungal alkaloids from *Mahonia fortunei* against pathogens of postharvest fruit**

Xiao-Na Wang^a,1^, Zhao-Jie Wang^a,1^, Yun Zhao^b^, Huan Wang^a^, Mei-Ling Xiang^a^, Yang-Yang Liu^a^, Li-Xing Zhao^a*^, Xiao-Dong Luo^a,b*^

*^a^ Key Laboratory of Medicinal Chemistry for Natural Resource, Ministry of Education and Yunnan Province, Yunnan Characteristic Plant Extraction Laboratory, School of Chemical Science and Technology, Yunnan University, Kunming, 650500, PR China*

*^b^ State Key Laboratory of Phytochemistry and Plant Resources in West China, Kunming Institute of Botany, Chinese Academy of Sciences Kunming, 650201, P. R. China*

__________________________________________________

* Corresponding author.

Tel.: +86-0871-65223177;

*E-mail address:* xdluo@ynu.edu.cn (X.-D. Luo); lixingzhao@ynu.edu.cn (L.-X. Zhao).

^1^ These authors contributed equally to this work.

1. Separation process and NMR information of 18 compounds

Fr. I (1.8 g) was separated on a silica gel column using a gradient of petroleum ether–acetone (4:1 → 1:1, v/v) and CHCl_3_-ethyl acetate (6:1, v/v) to afford compound **1** (10.0 mg). Fr. IV (22.4 g) was subjected to medium-pressure liquid chromatography (MPLC) over an MCI column with a gradient of MeOH-H_2_O (30:70 → 100:0, v/v) followed by silica gel column using a gradient of CHCl_3_-MeOH (100:1 → 20:1, v/v) to produce compounds **6** (16 mg), **7** (34.0 mg) and **8** (5.0 mg), **9** (35.0 mg), **10** (7.0 mg), **11** (15.0 mg) and **12** (6 mg). Fr. V (10.3 g) was chromatographed on a silica gel column using a gradient of CHCl_3_-MeOH (15:1 → 7:1, v/v) and ethyl acetate-MeOH (8:1, v/v) to produce compounds **13** (100.0 mg), **14** (17.0 mg), **15** (11.0 mg), **16** (10.0 mg), **17** (8.0 mg) and **18** (36.0 mg). Fr. VI was subjected to MPLC on an MCI column and chromatographed on a silica gel column using a gradient of CHCl_3_-MeOH (100:1, v/v) added with 1% ammonia water to produce compounds **2** (20 mg), **3** (8 mg), **4** (8 mg) and **5** (15 mg).

***Sinotumine I* (1)** [1]**:** C_20_H_19_NO_5_; ^1^H NMR (400 MHz, CDCl_3_) *δ*_H_: 7.23 (1H, d, *J* = 8.5 Hz, H-11), 7.20 (1H, d, *J* = 8.6 Hz, H-12), 7.14 (1H, s, H-1), 6.73 (1H, s, H-4), 6.66 (1H, s, H-13), 4.22 (1H, t, *J* = 5.8 Hz, H-6), 3.94 (3H, s, 9-OCH_3_), 3.92(3H, s, 2-OCH_3_), 3.87 (3H, s, 10-OCH_3_), 2.81 (1H, t, *J* = 6.3 Hz, H-5); ^13^C NMR (100 MHz, CDCl_3_) *δ*_C_: 160.3 (s, C-8), 151.3 (s, C-10), 149.5 (s, C-9), 146.8 (s, C-3), 146.1 (s, C-2), 135.9 (s, C-13a), 132.5 (s, C-12a), 129.3 (s, C-4a), 122.1 (d, C-12), 122.1 (s, C-1a), 119.3 (s, C-8a), 119.0 (d, C-11), 113.7 (d, C-4), 106.9 (d, C-1), 100.7 (d, C-13), 61.6 (q, 9-OCH_3_), 56.9 (q, 10-OCH_3_), 56.3 (q, 2-OCH_3_), 39.5 (t, C-6), 28.0 (t, C-5); positive ESI-MS *m/z* 354 [M + H]^+^.

***Limacine* (2)** [2]**:** C_37_H_40_N_2_O_6_; ^1^H NMR (400 MHz, CD_3_OD) *δ*_H_: 7.34 (1H, dd, *J* = 8.6, 2.3 Hz, H-14´), 7.01 (1H, dd, *J* = 8.2, 2.7 Hz, H-13´), 6.85 (1H, dd, *J* = 8.3, 1.2 Hz, H-11´), 6.77 (1H, dd, *J* = 8.2, 2.0 Hz, H-10´), 6.70 (1H, s, H-5´), 6.56 (1H, dd, *J* = 8.3, 2.1 Hz, H-14), 6.44 (1H, d, *J* = 8.2 Hz, H-13), 6.40 (1H, s, H-10), 6.36 (1H, s, H-5), 5.94 (1H, s, H-8´), 3.98 (1H, dd, *J* = 10.0, 1.5 Hz, H-1), 3.84 (3H, s, 6-OCH_3_), 3.72 (3H, s, 12-OCH_3_), 3.63 (1H, d, *J* = 1.0 Hz, H-1´), 3.34 (3H, s, 6´-OCH_3_), 3.22 (2H, dd, *J* = 12.0, 5.5 Hz, H-*α*´), 2.96 (4H, m, H-3´, 3), 2.84 (4H, m, H-4´, 4), 2.68 (2H, m, H-*α*), 2.52 (3H, s, *N*´-CH_3_), 2.20 (3H, s, *N*-CH_3_); ^13^C NMR (100 MHz, CD_3_OD) *δ*_C_: 154.6 (s, C-12´), 150.2 (s, C-11), 149.8 (s, C-6´), 147.6 (s, C-6), 147.3 (s, C-12), 143.9 (s, C-8), 143.9 (s, C-7´), 135.10 (s, C-7), 135.0 (s, C-9´), 134.3 (s, C-9), 132.2 (d, C-10´), 130.2 (s, C-8a´), 128.6 (t, C-4´), 128.1 (d, C-14´), 123.7 (s, C-8a), 122.9 (s, C-4a), 121.5 (d, C-13´), 121.3 (d, C-8´), 120.4 (d, C-11´), 115.9 (d, C-10), 111.9 (d, C-5´), 111.7 (d, C-13), 105.5 (d, C-5), 63.5 (d, C-14), 61.4 (d, C-l), 55.4 (q, 6-OCH_3_), 55.2 (q, 12-OCH_3_), 55.0 (q, 6´-OCH_3_), 45.0 (q, *N*´-CH_3_), 44.3 (t, C-3), 41.7 (q, *N*-CH_3_), 41.2 (d, C-1´), 37.7 (t, C-*α*), 35.6 (t, C-*α*´), 24.9 (t, C-3´), 23.2 (t, C-4); positive ESI-MS *m/z* 609 [M + H]^+^.

***Palmatine* (3)** [3]**:** C_21_H_22_NO_4_^+^; ^1^H NMR (400 MHz, DMSO-*d_6_*) *δ*_H_: 9.83 (1H, s, H-8), 8.99 (1H, s, H-13), 8.15 (1H, d, *J* = 9.3 Hz, H-11), 7.97 (1H, d, *J* = 8.7 Hz, H-12), 7.66 (1H, s, H-1), 6.03 (1H, s, H-4), 4.89 (2H, t, *J* = 6.0 Hz, H-6), 4.04 (3H, s, 9-OCH_3_), 4.01 (3H, s, 10-OCH_3_), 3.88 (3H, s, 3-OCH_3_), 3.81 (3H, s, 2-OCH_3_), 3.17 (2H, t, *J* = 7.2 Hz, H-5); ^13^C NMR (100 MHz, DMSO-*d_6_*) *δ*_C_: 152.0 (s, C-3), 150.7 (s, C-9), 149.2 (s, C-2), 146.0 (d, C-8), 144.1 (s, C-10), 138.2 (s, C-14), 133.6 (s, C-8a), 129.1 (s, C-4a), 127.3 (d, C-12), 123.9 (d, C-11), 121.9 (s, C-12a), 120.4 (d, C-13), 119.4 (s, C-14a), 111.8 (d, C-4), 109.2 (d, C-1), 62.4 (q, 9-OCH_3_), 57.5 (q, 10-OCH_3_), 56.6 (q, 3-OCH_3_), 56.3 (q, 2-OCH_3_), 55.9 (t, C-6), 26.5 (t, C-5); positive ESI-MS *m/z* 352 [M]^+^.

***Reticuline* (4)** [4]**:** C_19_H_23_NO_4_; ^1^H NMR (400 MHz, CDCl_3_) *δ*_H_: 6.77 (1H, d, *J* = 2.0 Hz, H-15), 6.73 (1H, d, *J* = 8.2 Hz, H-12), 6.59 (1H, dd, *J* = 2.1, 8.1 Hz, H-11), 6.54 (1H, s, H-5), 6.39 (1H, s, H-8), 3.85 (3H, s, 13-OCH_3_), 3.84 (3H, s, 14-OCH_3_), 3.68 (1H, dd, *J* = 6.3, 6.3 Hz, H-1), 3.17 (1H, ddd, *J* = 16.1, 6.5, 3.6 Hz, H-3a), 3.16 (1H, m, H-4a), 3.03 (1H, dd, *J* = 14.1, 6.2 Hz, H-9a), 2.77 (1H, dd, *J* = 14.4, 6.0 Hz, H-9b), 2.74 (1H, m, H-3b), 2.58 (1H, m, H-4b), 2.46 (3H, s, *N-*CH_3_); ^13^C NMR (100 MHz, CDCl_3_) *δ*_C_: 145.3 (s, C-6), 145.1 (s, C-14), 145.0 (s, C-13), 143.4 (s, C-7), 133.2 (s, C-10), 130.2 (s, C-8a), 125.3 (s, C-4a), 120.9 (d, C-11), 115.6 (d, C-15), 113.7 (d, C-8), 110.6 (d, C-5), 110.5 (d, C-12), 64.5 (d, C-1), 55.9 (q, 14-OCH_3_), 55.9 (q, 13-OCH_3_), 46.8 (t, C-3), 42.4 (q, *N*-CH_3_), 41.0 (t, C-9), 25.0 (t, C-4); positive ESI-MS *m/z* 330 [M + H]^+^.

***Berbamine* (5)** [5]**:** C_37_H_40_N_2_O_6_; ^1^H NMR (400 MHz, CDCl_3_) *δ*_H_: 7.19, (1H, dd, *J* = 8.4, 2.3 Hz, H-10´), 7.02 (1H, dd, *J* = 8.1, 2.6 Hz, H-11´), 6.70 (1H, d, *J* = 8.7 Hz, H-13), 6.76 (1H, d, *J* = 7.4 Hz, H-14), 6.53 (1H, dd, *J* = 8.2, 2.7 Hz, H-13´), 6.51 (1H, s H-5´), 6.35 (2H, m, H-10, 14´), 6.19 (1H, s, H-5), 5.90 (1H, bs, H-8´), 3. 80 (2H, m, H-1, 1´), 3.75 (3H, s, 6-OCH_3_), 3.50 (3H, s, 6´-OCH_3_), 3.36 (2H, m, H-3, 9´), 3.36 (1H, m, H-3´), 3.19 (3H, m, H-9´, 4´, 3´), 3.03 (3H, s, 7´-OCH_3_), 2.95 (1H, d, *J* = 13.8 Hz, H-9), 2.79 (3H, m, H-9, 3, 4), 2.54 (1H, m, H-4´), 2.39 (1H, m, H-4), 2.50 (3H, s, 2´-*N*CH_3_), 2.10 (3H, s, 2-*N*CH_3_); ^13^C NMR (100 MHz, CDCl_3_) *δ*_C_: 152.9 (s, C-12´), 150.8 (s, C-6), 148.9 (s, C-6´), 147.1 (s, C-8), 146.6 (s, C-11), 142.9 (s, C-12), 142.5 (s, C-7´), 135.9 (s, C-7), 134.4 (s, C-9a, 9a´), 131.3 (d, C-10´), 131.2 (t, C-14´), 129.2 (s, C-4a), 127.6 (s, C-4a´), 126.1 (s, C-8a´), 122.5 (d, C-14), 122.5 (d, C-11´), 120.7 (s, C-8a), 120.4 (d, C-13´), 119.5 (d, C-8´), 114.4 (d, C-10), 113.9 (d, C-13), 110.2 (d, C-5´), 104.4 (d, C-5), 62.6 (d, C-1), 62.6 (d, C-1´), 59.5 (q, 7-OCH_3_), 54.7 (q, 6-OCH_3_), 54.5 (q, 6´-OCH_3_), 44.7 (t, C-3, 3´), 41.7 (q, 2-*N*CH_3_), 41.6 (q, 2´-*N*CH_3_), 37.4 (s, C-9´), 36.7 (s, C-9), 28.7 (t, C-4´), 24.2 (t, C-4); positive ESI-MS *m/z* 609 [M + H]^+^.

***Obamegine* (6)** [5]**:** C_36_H_38_N_2_O_6_; ^1^H NMR (400 MHz, CDCl_3_) *δ*_H_: 7.29, (1H, dd, *J* = 8.6, 2.2 Hz, H-10´), 7.00 (1H, dd, *J* = 8.2, 2.6 Hz, H-11´), 6.74 (2H, dd, *J* = 8.6, 2.4 Hz, H-13, 14), 6.65 (1H, dd, *J* = 8.2, 2.7 Hz, H-13´), 6.68 (1H, s H-5´), 6.36 (1H, dd, *J* = 8.0, 2.1 Hz, H-14´), 6.29 (1H, s, H-10), 6.18 (1H, s, H-5), 3. 99 (1H, dd, *J* = 11.0, 2.1 Hz, H-1), 3.85 (7H, d, *J* = 3.2 Hz, H-1´, 6-OCH_3_, 6´-OCH_3_), 3.47 (2H, m, H-3, 9´), 3.30 (1H, m, H-3´), 3.20 (1H, m, H-9´), 2.91 (2H, m, H-4´, 3´), 2.85 (2H, d, *J* = 3.8 Hz, H-9), 2.74 (3H, m, H-3, 4, 4´), 2.62 (1H, m, H-4), 2.45 (3H, s, 2´-*N*CH_3_), 2.25 (3H, s, 2-*N*CH_3_); ^13^C NMR (100 MHz, CDCl_3_) *δ*_C_: 153.8 (s, C-12´), 149.2 (s, C-6), 148.7 (s, C-6´), 146.1 (s, C-8), 145.9 (s, C-11), 143.0 (s, C-12), 142.7 (s, C-7´), 135.2 (s,C-7), 133.4 (s, C-9a´), 132.0 (d, C-10´), 131.0 (d, C-14´), 129.3 (s, C-9a), 129.0 (s, C-4a´), 127.7 (s, C-8a´), 123.3 (d, C-14), 121.9 (d, C-11´), 121.8 (s, C-8a), 121.1 (d, C-13´), 120.8 (d, C-8´), 120.6 (s, C-4a), 114.1 (d, C-10), 111.3 (d, C-13), 110.5 (d, C-5´), 106.5 (d, C-5), 63.6 (d, C-1´), 59.2 (d, C-1), 55.1 (q, 6-OCH_3_), 55.0 (q, 6´-OCH_3_), 44.1 (t, C-3´), 43.0 (t, C-3), 41.3 (q, 2´-*N*CH_3_), 41.2 (q, 2-*N*CH_3_), 37.9 (t, C-9´), 37.6 (t, C-9), 23.5 (t, C-4´), 21.8 (t, C-4); positive ESI-MS *m/z* 595 [M + H]^+^.

***Fenfangjine A* (7)** [6]: C_38_H_42_O_7_N_2_; ^1^H NMR (400 MHz, CDCl_3_) *δ*_H_: 7.27 (1H, dd, *J* = 8.1, 2.2 Hz, H-14´), 6.82 (4H, d, *J* = 8.3 Hz, H-13´, H-11´, H-13, H-10), 6.71 (1H, dd, *J* = 8.2, 2.3 Hz, H-14), 6.45 (1H, s, H-10´), 6.35 (1H, s, H-5´), 6.22 (1H, s, H-5), 5.97 (1H, s, H-8´), 4.84 (1H, d, *J* = 9.4 Hz, H-1), 3.91 (3H, s, 12-OCH_3_), 3.76 (3H, s, 6-OCH_3_), 3.52 (3H, s, 6´-OCH_3_), 3.45 (3H, m, 7-OCH_3_), 3.02 (3H, s, 2-*N*CH_3_), 2.57 (3H, s, 2´-*N*CH_3_); ^13^C NMR (100 MHz, CDCl_3_) *δ*_C_: 154.5 (s, C-12´), 152.8 (s, C-6), 150.2 (s, C-12), 149.9 (s, C-6´), 148.7 (s, C-8), 147.2 (s, C-11), 142.6 (s, C-7´), 142.6 (s, C-7), 136.9 (s, C-9´), 136.9 (s, C-9), 132.2 (s, C-4a), 132.1 (d, C-14´), 130.1 (d, C-10´), 129.5 (s, C-4a´), 127.6 (s, C-8a´), 125.9 (s, C-8a), 122.8 (d, C-14), 121.3 (s, C-11´), 120.6 (d, C-13´), 120.1 (d, C-8´), 116.8 (s, C-11), 111.8 (d, C-13), 111.8 (d, C-5´), 104.7 (d, C-5), 77.2 (d, C-1), 63.1 (d, C-1´), 60.8 (q, 2-*N*CH_3_), 59.9 (q, 7-OCH_3_), 57.1 (t, C-3), 56.2 (q, 6´-OCH_3_), 55.8 (q, 12-OCH_3_), 55.5 (q, 6-OCH_3_), 45.7 (t, C-3´), 42.7 (t, C-*α*), 42.7 (q, 2´-*N*CH_3_), 37.3 (t, C-*α*´), 25.7 (t, C-4), 25.0 (t, C-4´); positive ESI-MS *m/z* 639 [M + H]^+^.

***N-allyllaurolitsine* (8)** [7]**:** C_21_H_23_NO_4_; ^1^H NMR (400 MHz, DMSO-*d_6_*) *δ*_H_: 7.80 (1H, s, H-11), 7.03 (1H, d, *J* = 1.3 Hz, H-8), 6.63 (2H, m, *N*-CH_2_CH=CH_2_), 6.38 (1H, dt, *J* = 16.0, 4.4 Hz, 2´*N*-CH_2_CH=CH_2_), 3.94 (3H, s, 10-OCH_3_), 3.68 (3H, s, 1-OCH_3_), 3.17 (2H, s, 1´*N*-CH_2_CH=CH_2_); ^13^C NMR (100 MHz, DMSO-*d_6_*) *δ*_C_: 147.7 (s, C-2), 145.5 (s, C-9), 144.8 (s, C-10), 143.3 (s, C-1), 133.3 (d, 2´*N*-CH_2_CH=CH_2_), 130.2 (s, C-3a), 129.6 (s, C-7a), 129.2 (s, C-1b), 127.5 (s, C-1a), 122.4 (s, C-11a), 121.6 (t, 3´*N*-CH_2_CH=CH_2_), 115.5 (d, C-8), 113.4 (d, C-3), 111.0 (d, C-11), 62.1 (q, 1-OCH_3_), 56.3 (s, C-6a), 55.9 (t, 1´*N*-CH_2_CH=CH_2_), 53.8 (q, 10-OCH_3_), 49.1 (t, C-5), 34.4 (t, C-7), 29.9 (t, C-4); positive ESI-MS *m/z* 354 [M + H]^+^.

***Isotetrandrine* (9)** [8]**:** C_38_H_42_N_2_O_6_; ^1^H NMR (400 MHz, CDCl_3_) *δ*_H_: 7.28 (1H, dd, *J* = 8.3, 1.7 Hz, H-10´), 7.10 (1H, dd, *J* = 8.0, 2.6 Hz, H-11´), 6.80 (2H, d, *J* = 8.4 Hz, H-13, H-14), 6.65 (1H, dd, *J* = 8.3, 2.5 Hz, H-10), 6.54 (1H, s, H-5´), 6.43 (2H, s, H-10, H-14´), 6.27 (1H, s, H-5), 5.98 (1H, s, H-8´), 3.91 (3H ,d, 12-OCH_3_), 3.75 (3H, s, 6-OCH_3_), 3.61 (3H, s, 6´-OCH_3_), 3.40 (1H, m, Ha-3´), 3.24 (1H, dd, *J* = 13.8, 8.8 Hz, Ha-3), 3.13 (3H, s, 7-OCH_3_), 2.26 (6H, s, 2-*N*CH_3_, 2´-*N*CH_3_); ^13^C NMR (100 MHz, CDCl_3_) *δ*_C_: 154.0 (s, C-12´), 151.9 (s, C-6), 149.9 (s, C-11), 149.6 (s, C-6´), 148.4 (s, C-8), 147.0 (s, C-12), 143.63 (s, C-7´), 137.1 (s, C-7), 135.3 (s, C-9,9´), 132.1 (d, C-14´), 130.2 (d, C-10´), 128.9 (s, C-4a, 4a´), 127.8 (s, C-8a´), 122.8 (d, C-14), 122.1 (d, C-13´), 121.7 (d, C-11´), 120.7 (s, C-8a), 119.8 (d, C-8´), 115.9 (d, C-10), 111.3 (d, C-13), 111.3 (d, C-5´), 105.6 (d, C-5), 64.2 (d, C-1´), 62.0 (d, C-1), 60.5 (q, 7-OCH_3_), 56.1 (q, 12-OCH_3_), 55.8 (q, 6´-OCH_3_), 55.6 (q, 6-OCH_3_), 46.1 (t, C-3, 3´), 42.9 (q, 2´-*N*CH_3_), 42.7 (q, 2-*N*CH_3_), 38.8 (t, C-15), 37.8 (t, C-15´), 25.7 (t, C-4´, 4); positive ESI-MS *m/z* 623 [M + H]^+^.

***Erysotrine* (10)** [9]**:** C_19_H_23_NO_3_; ^1^H NMR (400 MHz, CDCl_3_) *δ*_H_: 7.07 (1H, dd, *J* = 8.0, 2.0 Hz, H-1), 6.79 (1H, s, H-14), 6.72 (1H, s, H-17), 6.26 (1H, d, *J* = 10.0 Hz, H-2), 6.16 (1H, bs, H-7), 4.01 (1H, m, H-3), 3.93 (3H, s, 16-OCH_3_), 3.92 (1H, m, H-8), 3.84 (3H, s, 15-OCH_3_), 3.81 (1H, s, 3-OCH_3_), 3.49 (1H, m, H-8), 3.11 (1H, m, H-10), 2.94 (1H, m, H-10), 2.76 (1H, m, H-11), 2.59 (1H, m, H-11), 2.50 (1H, m, H-4), 1.59 (1H, m, H-4); ^13^C NMR (100 MHz, CDCl_3_) *δ*_C_: 149.6 (s, C-16), 146.8 (s, C-15), 144.2 (s, C-6), 131.6 (d, C-2), 130.1 (s, C-13), 124.7 (s, C-12), 123.7 (d, C-1), 123.4 (d, C-7), 111.7 (d, C-17), 111.3 (d, C-14), 77.2 (d, C-3), 67.9 (s, C-5), 56.2 (t, C-8), 56.1 (q, 15-OCH_3_), 56.1 (q, 16-OCH_3_), 56.0 (q, 3-OCH_3_), 42.7 (t, C-10), 39.4 (t, C-4), 25.2 (t, C-11); positive ESI-MS *m/z* 314 [M + H]^+^.

***Fenfangjine P* (11)** [10]**:** C_37_H_40_O_7_N_2_; ^1^H NMR (400 MHz, CDCl_3_) *δ*_H_: 7.31 (1H, dd, *J* = 8.1, 2.1 Hz, H-14´), 7.05 (1H, dd, *J =* 8.2, 2.3 Hz, H-13´), 6.82 (1H, d, *J* = 8.3 Hz, H-13), 6.65 (3H, m, H-14, 11´, 10), 6.51 (1H, s, H-5´), 6.39 (1H, s, H-5), 6.31 (1H, dd, *J* = 8.6, 2.2 Hz, H-10´), 6.11 (1H, s, H-8´), 4.87 (1H, brd, *J* = 10.0 Hz, H-1), 3.93 (3H, s, 12-OCH_3_), 3.86 (1H, m, H-1´), 3.78 (3H, s, 6-OCH_3_), 3.78 (3H, s, 6´-OCH_3_), 3.66 (1H, m, H-3), 3.46 (3H, m, H-4, 3, 3´), 3.27 (2H, m, H-8´, *α*), 3.07 (3H, s, 2-*N*CH_3_), 2.81 (7H, m, H-3´, H-4´, H-4, H-*α*´, H-*α*), 2.54 (3H, s, 2´-*N*CH_3_); ^13^C NMR (100 MHz, CDCl_3_) *δ*_C_: 154.2 (s, C-12´), 150.6 (s, C-11), 149.3 (s, C-6´), 148.3 (s, C-12), 147.8 (s, C-6), 142.9 (s, C-7´), 142.2 (s, C-8), 135.8 (s, C-9´), 135.5 (s, C-7), 132.1 (d, C-10´), 131.0 (s, C-9), 130.8 (d, C-14´), 129.6 (s, C-8a´), 121.8 (d, C-14), 121.8 (d, C-11´), 121.7 (d, C-13´), 121.6 (s, C-4a), 120.9 (s, C-8a), 118.2 (d, C-8´), 115.6 (d, C-10), 112.0 (d, C-5´), 111.7 (d, C-13), 105.9 (d, C-5), 74.9 (d, C-1), 63.8 (d, C-1´), 58.7 (q, 2-*N*CH_3_), 56.5 (t, C-3), 56.2 (q, 6-OCH3), 56.1 (q, 12-OCH_3_), 56.0 (q, 6´-OCH_3_), 46.3 (t, C-3´), 43.0 (t, C-*α*), 42.9 (q, 2´-*N*CH_3_), 38.1 (t, C-*α*´), 25.8 (t, C-4´), 24.6 (t, C-4); positive ESI-MS *m/z* 625 [M + H]^+^.

***Coptisonine* (12)** [11]**:** C_19_H_14_N^+^O_5_; ^1^H NMR (400 MHz, CDCl_3_) *δ*_H_: 12.33 (1H, br. s, OH), 8.37 (1H, d, *J* = 5.7 Hz, H-6), 7.55 (1H, d, *J* = 5.3 Hz, H-5), 7.31 (1H, s, H-1), 7.13 (1H, d, *J* = 9.2 Hz, H-12), 7.09 (1H, s, H-4), 6.34 (1H, d, *J* = 9.2 Hz, H-11), 6.05 (2H, s, 2, 3-OCH_2_O), 3.86 (3H, s, 9-OCH_3_), 3.85 (3H, s, 10-OCH_3_); ^13^C NMR (100 MHz, CDCl_3_) *δ*_C_: 197.9 (s, C-13), 158.3 (s, C-10), 157.4 (s, C-8), 153.1 (s, C-14), 150.3 (s, C-3), 148.4 (s, C-2), 139.5 (d, C-6), 136.1 (s, C-9), 134.6 (s, C-4a), 129.6 (d, C-12), 122.5 (s, C-14a), 120.9 (d, C-5), 114.0 (s, C-12a), 102.3 (d, C-11), 101.8 (d, C-4), 100.9 (d, C-1), 100.9 (q, 2, 3-OCH_2_O), 59.8 (q, 9-OCH_3_), 55.2 (q, 10-OCH_3_); positive ESI-MS *m/z* 336 [M]^+^.

***Berberine* (13)** [3]**:** C_20_H_18_NO_4_^+^; ^1^H NMR (400 MHz, DMSO-*d_6_*) *δ*_H_: 9.90 (1H, s, H-8), 8.96 (1H, s, H-13), 8.20 (1H, d, *J* = 9.1 Hz, H-11), 8.01 (1H, d, *J* = 9.1 Hz, H-12), 7. 80 (1H, s, H-l), 7.09 (1H, s, H-4), 6.17 (2H, s, OCH_2_O), 4.94 (2H, m, H-6), 4.09 (3H, s, 9-OCH_3_), 4.07 (3H, s, 10-OCH_3_), 3.19 (2H, m, H-5); ^13^C NMR (100 MHz, DMSO-*d_6_*) *δ*_C_: 150.9 (s, C-3), 150.3 (s, C-10), 148.2 (s, C-2), 146.0 (d, C-8), 144.2 (s, C-9), 138.0 (s, C-14), 133.5 (s, C-12a), 131.2 (s, C-4a), 127.2 (d, C-11), 124.0 (d, C-12), 121. 9 (s, C-8a), 120.9 (d, C-13), 120.7 (s, C-14a), 108.9 (d, C-4), 105.9 (d, C-1), 102.57 (t, OCH_2_O), 62.4 (q, 9-OCH_3_), 57.5 (q, 10-OCH_3_), 55.7 (t, C-6), 26.8 (t, C-5); positive ESI-MS *m/z* 336 [M]^+^.

***Papaverine* (14)** [12]**:** C_20_H_21_NO_4_; ^1^H NMR (400 MHz, DMSO-*d_6_*) *δ*_H_: 7.20 (1H, m, H-4), 7.14 (1H, s, H-8), 6.75 (2H, d, *J* = 8.5 Hz, H-5, 5´), 6.64 (1H, s, H-2´), 6.38 (2H, m, H-2´, 6´), 3.84 (3H, s, 6-OCH_3_), 3.69 (3H, s, 3-OCH_3_), 3.45 (3H, s, 3´-OCH_3_), 2.94 (3H, s, 4´-OCH_3_); ^13^C NMR (100 MHz, DMSO-*d_6_*) *δ*_C_: 154.5 (s, C-1), 152.7 (s, C-6), 150.1 (s, C-7), 148.6 (s, C-3´), 147.2 (d, C-4´), 142.6 (s, C-3), 132.1 (s, C-4a), 129.5 (s, C-1´), 122.7 (d, C-8a), 121.3 (d, C-6´), 120.1 (s, C-4), 111.8 (d, C-2´), 110.7 (s, C-5´), 104.7 (d, C-5), 104.7 (d, C-8), 56.7 (q, 6-OCH_3_), 55.8 (q, 7-OCH_3_), 55.5 (q, 3´-OCH_3_), 55.4 (q, 4´-OCH_3_), 42.7 (t, C-1a); positive ESI-MS *m/z* 340 [M + H]^+^.

***N-methycoclaurine* (15)** [13]**:** C_18_H_21_NO_3_; ^1^H NMR (400 MHz, CDCl_3_) *δ*_H_: 6. 92 (2H, d, *J* = 4.2 Hz, H-2´, 6´), 6.90 (2H, d, *J* = 3.4 Hz, H- 3´, 5´), 6.59 (1H, s, H-5), 6.54 (1H, s, H-8), 3.83 (3H, s, OCH_3_), 3.71 (2H, m, H-1), 3.22 (1H, m, H-11b), 3.01 (1H, m, H-3b), 2.81 (1H, m, H-3a), 2.80 (1H, m, H-4a), 2.78 (1H, m, H-4b), 2.60 (1H, m, H-11a), 2.45 (3H, s, *N*-CH_3_); ^13^C NMR (100 MHz, CDCl_3_) *δ*_C_: 154.8 (s, C-4´), 145.5 (s, C-6), 143.4 (s, C-7), 130.3 (s, C-9), 130.0 (d, C-2´, 6´), 129.6 (s, C-1´), 124.7 (s, C-10), 115.6 (d, C-3´, 5´), 114.0 (d, C-5), 110.8 (d, C-8), 64.7 (d, C-1), 55.8 (q, OCH_3_), 46.0 (t, C-3), 41.8 (q, *N*-CH_3_), 40.5 (t, C-11), 24.2 (t, C-4); positive ESI-MS *m/z* 300 [M + H]^+^.

***(+)-4-hydroxysarcocapnine* (16)** [14]**:** C_20_H_23_NO_5_; ^1^H NMR (400 MHz, CDCl_3_) *δ*_H_: 7.19 (1H, d, *J* = 7.4 Hz, H-5), 6.82 (2H, m, H-6, 2´), 6.67 (1H, dd, *J* = 2.5, 7.8 Hz, H-3´), 4.40 (1H, bs, H-4), 4.12 (1H, q, H-1), 3.93 (3H, s, 5´-OCH_3_), 3.76 (3H, s, 4´-OCH_3_), 3.63 (3H, s, 6-OCH_3_), 3.31 (1H, d, *J* = 9.5 Hz, H-a), 3.07 (1H, d, *J* = 10.0 Hz, H-3a), 2.99 (1H, d, *J* = 13.4, H-3b), 2.84 (1H, d, *J* = 12.3 Hz, H-a), 2.23 (3H, s, *N*-CH_3_); ^13^C NMR (100 MHz, CDCl_3_) *δ*_C_: 151.8 (s, C-7), 151.6 (s, C-4´), 149.3 (s, C-6´), 144.7 (s, C-7a), 141.5 (s, C-5´), 128.9 (s, C-1a), 123.7 (s, C-4a), 123.1 (d, C-5), 119.6 (d, C-2´), 110.9 (d, C-6), 105.8 (d, C-3´), 67.2 (d, C-1), 60.2 (d, C-4), 59.3 (q, 5´-OCH_3_), 56.1 (q, 4´-OCH_3_), 55.8 (t, C-3), 55.7 (q, 7-OCH_3_), 42.6 (q, *N*-CH_3_), 37.8 (t, C-a); positive ESI-MS *m/z* 358 [M + H]^+^.

***Coclaurine* (17)** [15]**:** C_17_H_19_NO_3_; ^1^H NMR (400 MHz, DMSO-*d_6_*) *δ*_H_: 7.04 (2H, d, *J* = 8.7 Hz, H-2´, 4´), 6.68 (2H, d, *J* = 8.3 Hz, H-3´, 5´), 6.64 (1H, s, H-5), 6.58 (1H, s, H-8), 3.85 (1H, t, *J* = 5.0 Hz, H-1), 3.72 (3H, s, OCH_3_); ^13^C NMR (100 MHz, DMSO-*d_6_*) *δ*_C_: 156.0 (s, C-4´), 146.4 (s, C-6), 144.7 (s, C-5), 131.7 (s, C-9), 130.7 (d, C-2´, 6´), 130.1 (s, C-1´), 126.0 (s, C-10), 115.5 (d, C-3´, 5´), 113.8 (d, C-5), 112.8 (d, C-8), 56.8 (d, C-1), 56.0 (q, O-CH_3_), 41.7 (t, C-3), 40.6 (t, C-11), 29.5 (d, C-4); positive ESI-MS *m/z* 286 [M + H]^+^.

***Jatrorrhizine* (18)** [3]**:** C_20_H_20_NO_4_^+^; ^1^H NMR (400 Hz, DMSO-*d_6_*) *δ*_H_: 9.85 (1H, s, H-8), 8.96 (1H, s, H-13), 8.20 (1H, d, *J* = 9.4 Hz, H-11), 8.01 (1H, d, *J* = 8.8 Hz, H-12), 7.70 (1H, s, H-1), 6.85 (1H, s, H-4), 4.91 (2H, t, *J* = 6.4 Hz, H-6), 4.10 (3H, s, 9-OCH_3_), 4.07 (3H, s, 10-OCH_3_), 3.95 (3H, s, 2-OCH_3_), 3.15 (2H, t, *J* = 6.2 Hz, H-5); ^13^C NMR (100 MHz, DMSO-*d_6_*) *δ*_C_: 150.6 (s, C-3, 10), 148.4 (s, C-4), 145.7 (s, C-8), 144.1 (s, C-9), 138.6 (s, C-14), 133.7 (s, C-12a), 129.4 (s, C-4a), 127.3 (d, C-11), 123.8 (d, C-12), 121.7 (s, C-8a), 119.9 (d, C-13), 118.2 (s, C-14a), 115.4 (d, C-4), 110.0 (d, C-1), 62.3 (q, 9-OCH_3_), 57.5 (q, 10-OCH_3_), 56.7 (t, C-6), 55.9 (q, 2-OCH_3_), 26.2 (t, C-5); positive ESI-MS *m/z* 338 [M]^+^.

1. Antifungal activity of small polar alkaloids from *M. fortunei*

Table S1 Antifungal activity of small polar alkaloids from *M. fortunei*

| *Botrytis cinerea* | | *Penicillium italicum* | |
| --- | --- | --- | --- |
| MIC  mg/L | MFC  mg/L | MIC  mg/L | MFC  mg/L |
| 200 | 400 | 200 | 400 |

1. *
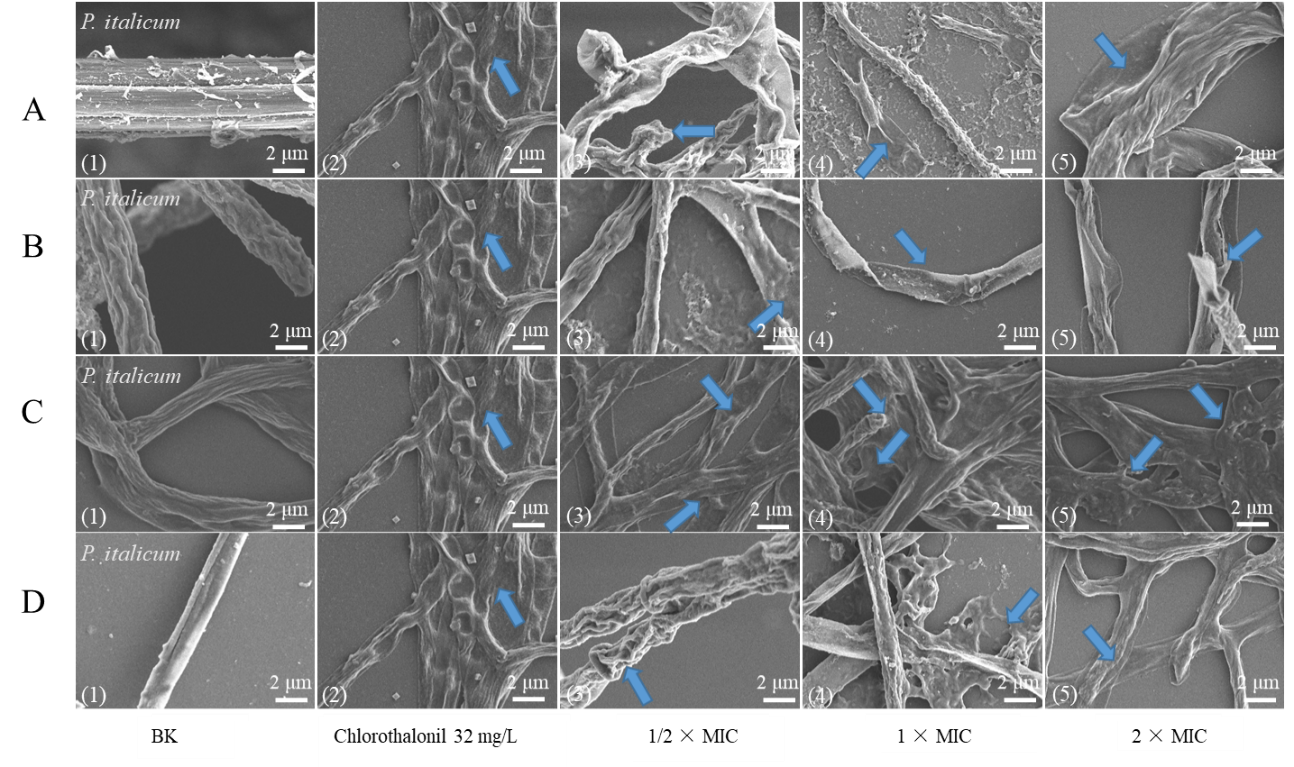
*The morphological changes of mycelium of *P. italicum*

**Figure S1.** Scanning electron microscope photos of *P. italicum* Untreated mycelium (A1-D1); mycelia treated with palmatine (A), berbamine (B), berberine (C) and jatrorrhizine (D) at 1/2 × MIC, 1 × MIC, 2 × MIC (A3-D5); mycelium treated with the positive control chlorothalonil at a concentration of 32 mg·L^-1^ (A2-D2). The blue arrow represents the observed morphological change.

4. Cytotoxicity assay.

The compounds berberine, jatrorrhizine and the positive control chlorothalonil were evaluated for their *in vitro* cytotoxic activity against HacaT cells. The results of the cytotoxicity test are shown in **Figure S2**. At a concentration of 16 μg/mL, the inhibitory rate of berberine on HacaT cells was 75%, the inhibitory rate of jatrorrhizine on HacaT cells was 35%, and the inhibitory rate of chlorothalonil on HacaT cells was as high as 95%. Therefore, the cytotoxicity of chlorothalonil is much greater than the cytotoxicity of the compounds berberine and jatrorrhizine. The same result was obtained at a concentration of 32 mg·L^-1^.

**Figure S2.** Cytotoxicity of compounds to HacaT cells

1. Lyu HN, Zeng KW, Cao NK, Zhao MB, Jiang Y, Tu PF. Alkaloids from the stems and rhizomes of *Sinomenium acutum* from the Qinling Mountains, China. Phytochemistry. 2018;156:241-249.
2. Lin LZ, Shieh HL, Angerhofer CK, Pezzuto JM, Ruangrungsi N. Cytotoxic and antimalarial bisbenzylisoquinoline alkaloids from *Cyclea barbata*. Journal of Natural Products. 1993;56:22-29.
3. Cheng X, Wang D, Jiang L, Yang D. DNA topoisomerase I inhibitory alkaloids from *Corydalis saxicola*. Chemistry & Biodiversity. 2008;5(7):1335-1344.
4. Cruz PEOd, Costa EV, Moraesa VRdS, Nogueiraa PCdL, Vendraminb ME. Chemical constituents from the bark of *Annona salzmannii* (Annonaceae). Biochemical Systematics and Ecology. 2011;39:872-875.
5. Hostalkova A, Marikova J, Opletal L, Korabecny J, Hulcova D, Kunes J, Novakova L, Perez DI, Jun D, Kucera T, Andrisano V, Siatka T, Cahlikova L. Isoquinoline alkaloids from *Berberis vulgaris* as potential lead compounds for the treatment of Alzheimer's disease. Journal of Natural Products. 2019;82(2):239-248.
6. Ogino T, Sato T, Sasaki H, Chin M, Mitsuhashi H. Four new bisbenzylisoquinoline alkaloids from the root of *Stephania tetrandra* (Fen-Fang Ji). Heterocycles. 1988;27(5):1149-1154.
7. Sulaiman SN, Mukhtar MR, Hadi AH, Awang K, Hazni H, Zahari A, Litaudon M, Zaima K, Morita H. Lancifoliaine, a new bisbenzylisoquinoline from the bark of *Litsea lancifolia*. Molecules. 2011;16(4):3119-3127.
8. Tanahashi T, Su Y, Nagakura N, Nayeshiro H. Quaternary isoquinoline alkaloids from *Stephania cepharantha*. Chemical and Pharmaceutical Bulletin. 2000;48(3):370-373.
9. Chawla AS, Chunchatprasert S, Jackson AH. Studies of erythrina alkaloids: VII-13C NMR spectral studies of some erythina alkaloids. Organic Magnetic Resonance. 1983;21(1):39-41.
10. Wang R, Liu Y, Shi G, Zhou J, Yu D. Bioactive bisbenzylisoquinoline alkaloids from the roots of *Stephania tetrandra*. Bioorganic Chemistry. 2020;98:103697.
11. Yang TC, Chao HF, Shi LS, Chang TC, Lin HC, Chang WL. Alkaloids from *Coptis chinensis* root promote glucose uptake in C2C12 myotubes. Fitoterapia. 2014;93:239-244.
12. Janssen RHAM, Lousberg RJJC, Wijkens P, Kruk C, Theuns HG. Assignment of 1H and 13C NMR resonances of some isoquinoline alkaloids. Phytochemistry. 1989;28(10):2833-2839.
13. Lee JK, Cho JG, Song MC, Yoo JS, Lee DY, Yang HJ, Han KM, Kim DH, Oh YJ, Jeong TS. Isolation of isoquinoline alkaloids from the tuber of *Corydalis turtschaninovii* and their inhibition activity on low density lipoprotein oxidation. Journal of the Korean Society for Applied Biological Chemistry. 2009;52(6):646-654.
14. Tojo E, Dominguez D, Castedo L. Alkaloids from *Sarcocapnos-Enneaphylla*. Phytochemistry. 1991;30(3):1005-1010.
15. Lei Y, Wu LJ, Bi D, Sun JW, Tu PF. Isolation and identification of chemical constituents from stems of *Miliusa balansae* Fin. et Gag. Journal of Shenyang Pharmaceutical University. 2009;26:104-107.
